# Undiscovered Biodiversity of the European Moss Flora: *Neodicranella hamulosa* (Aongstroemiaceae), a New Genus and Species from SW Portugal

**DOI:** 10.3390/plants10112289

**Published:** 2021-10-25

**Authors:** Ronald D. Porley, Vladimir Fedosov, Vítězslav Plášek, Alina Fedorova

**Affiliations:** 1 Cerca dos Pomares, CxP 409M, 8670-052 Aljezur, Portugal; 2 Faculty of Biology, Lomonosov Moscow State University, 119234 Moscow, Russia; fedosov_v@mail.ru; 3 Botanical Garden-Institute, FEB RAS, Makovskogo Street 142, 690024 Vladivostok, Russia; 4 Department of Botany, University of Ostrava, Chittussiho 10, 710 00 Ostrava, Czech Republic; vitezslav.plasek@osu.cz; 5 Tsitsin Main Botanical Garden of RAS, 127276 Moscow, Russia; alina_77777@mail.ru

**Keywords:** algarve, bayesian phylogenetic analysis, *Dicranella*, European moss endemic, *Nad5*, *rps4*, serra de monchique, taxonomy, *trnL-trnF*

## Abstract

A new genus is described to accommodate *Neodicranella hamulosa*, a novel species resolved in the family Aongstroemiaceae, from the Monchiquense district in SW Portugal. Characterized by its small size, erect spreading to subsecund non-sheathing leaves, plane bistratose leaf margins, and rhizoidal gemmae with slightly protruberant cells, it differs from all other European Dicranellaceae in the uniquely patterned distal peristome segments with backward-pointing papillae resembling hooked barbs. The species appears to be endemic to the sub-Mediterranean bioclimatic zone, in wooded biomes where humidity remains relatively high throughout the year. Morphological and molecular data strongly support the singularity of this new taxon. The species is illustrated by photomicrographs and SEM, and its ecology and conservation are discussed.

## 1. Introduction

Present on all continents, the genus *Dicranella* (Müll.Hal.) Schimp. (syn. *Anisothecium*), in the broader sense, is estimated to comprise somewhere between 158 (including *Anisothecium*) [1] and 162 species [2], while the Tropicos database (http://legacy.tropicos.org/NameSearch.aspx, accessed 6 September 2021) lists 175 accepted names. However, only about 50 species of *Dicranella* have been thoroughly described in revisions, floras, and monographs, whereas for some 100 or more species, there is no information available since first described (or post-1962), and therefore, the group is urgently in need of taxonomic revision [2,3]; there are only a few regional revisions [4,5] or studies of individual species [6], and only a small proportion of *Dicranella* s.l. has been sequenced [7]. In the past, some authors have recognized the genus *Anisothecium* (species of *Dicranella* possessing stomata, peristome with a relatively low basal membrane and with an annulus [8]), but this concept is not currently followed [4,9]. *Dicranella* in North America [9] is well understood, with 10 species (excluding *Dicranella palustris* (Dicks.) Crundw. & E. F. Warb., now placed in the genus *Diobelonella* [10]), and is well understood in Europe, where 12 species are accepted [11]. Nine species are listed for the Iberian Pensinsula [12], with six species reported for Portugal [12,13,14,15]: *D. heteromalla* (Hedw.) Schimp., *D. howei* Renauld & Cardot, *D. rufescens* (Dicks.) Schimp., *D. staphylina* H. Whitehouse, *D. subulata* (Hedw.) Schimp., and *D. varia* (Hedw.) Schimp.

During the ongoing field work by R.D.Porley on the Serra de Monchique in Algarve and on autochotonous *Quercus faginea* Lam. woodlands in the Monchiquense district [16], an enigmatic plant was found that appeared to belong to the Dicranellaceae M. Stech, but its identity was uncertain. It was suggestive of *Dicranella varia* (Hedw.) Schimp., but the plain leaf margin, narrow nerve, and relatively long mid-leaf laminal cells seemed to preclude that species. Material was nevertheless provisionally allocated to this species or put aside as identification pending. Over a period of 4 years, the same plant was collected from five different localities, but a connection was not made until 2020, when it was gathered from the Vale de Cova da Serra. A closer scrutiny of this specimen revealed a striking feature that was previously overlooked: the distal part of the peristome teeth is covered with distinctive backward-pointing papillae, reminiscent of hooked barbs. The earlier collections of *Dicranella* cf. *varia* from the Serra de Monchique were re-examined and found to show an identical peristome structure. Other European *Dicranella* peristomes were subsequently examined by light microscopy, and none were found to show this distinctive ornamentation. A study of the gametophyte of *Dicranella staphylina* H. Whitehouse and descriptions of *D. campylophylla* (Taylor) A. Jaeger [7] also clearly rejected the possibility that they are conspecific with the Portuguese plants. Porley then sent samples of the Portuguese plant to Ryszard Ochyra (Kraków), who shared the view that it may be a new species of *Dicranella*, and suggested the material be sent to V. Fedosov, who had previously studied Holarctic *Dicranella* using molecular phylogenetic methods. Recent molecular phylogenetic studies, largely focused on Holarctic *Dicranella* species [4], revealed deep polyphyly of the genus in its currently accepted sense. Species assigned to *Dicranella* were found in several groups dispersed throughout the phylogenetic tree of haplolepideous mosses, signifying possible impending major changes in nomenclature. The phylogenetic study by Bonfim-Santos et al. [4] did not provide a critical taxonomic treatment, as much more extensive sampling of taxa is needed; rather it aimed to provide a baseline for future studies on the phylogenetic relationships and circumscriptions of the Aongstroemiaceae and Dicranellaceae. All European *Dicranella* species have been sequenced in the recent study of Bonfim-Santos et al. [4], aside from *D. humilis* R. Ruthe., but this species was subsequently sequenced by Fedosov et al. (in press) and in this analysis. The principal aim of this study is to elucidate the identity of the unknown Portuguese plants by (i) morphological analyses and (ii) molecular analyses by comparing the sequences of the Portuguese plants with the DNA sequence data obtained by Bonfim-Santos et al. [4].

## 2. Materials and Methods

### 2.1. Morphological Study

The novel Portuguese plants were examined by standard light microscopy and SEM photomicrography, together with field observations. Macro photos were taken with Nikon D300s using the type material, and Microscope Olympus BX53 was used for all microscopic images. Detailed SEM images were taken on scanning microscopes JEOL JSM-6610LV and JEOL JSM 6380, coated by gold without any additional preparation. All micro and SEM images are scaled, and the sizes are explained in the legends of the figures. Other *Dicranella* were also examined with an emphasis on the peristome structure (where available), including all European species and *Diobelonella palustris* (Dicks.) Ochyra.

### 2.2. Molecular Phylogenetic Analysis

For the molecular phylogenetic study, we used a combination of two plastid loci *rps4* and *rps4-trnS* spacers, the *trnL-F* region and mitochondrial *Nad5*, which was successfully used for the study of the backbone phylogeny of the genus *Dicranella* [4]. This not only allowed a series of previously studied *Dicranella* accessions to be used but also enabled new specimens to be incorporated in the phylogenetic tree, including three specimens of the unknown Portuguese species (see Appendix A). The laboratory protocol followed Fedosov et al. [17,18]. After the initial phylogenetic analyses, the position of the target species was identified and consequently the dataset was largely focused on the Aongstroemiaceae clade *sensu* (Bonfim-Santos et al. [4]), with the addition of a suite of outgroup taxa. In addition, included in the molecular analysis was a specimen of *Dicranella humilis* R. Ruthe, of which the gametophyte is morphologically similar to that of the Portuguese plants, and *Diobelonella palustris* (Dicks.) Ochyra, the latter identified by the BLAST algorithm (https://blast.ncbi.nlm.nih.gov/Blast.cgi [accessed 8 May 2021]) as having the most similar *rps4* sequence.

The sequences were aligned manually using BioEdit [19]. The indel data in all analyses were scored using the simple indel coding approach [20] using SeqState 1.4.1. [21]. In the single-gene analyses, *rps4* was divided into four partitions, which corresponded to the *trnS-rps4* spacer and three codon positions of the coding part of the gene; *trnL-F* was divided into four partitions for the *trnL* exon, *trnL* intron, *trnL-F* spacer, and *trnF* gene correspondingly; *Nad5* was divided into two partitions, which corresponded to the exon and the intron. In the combined dataset, all plastid and mitochondrial nucleotide data were combined in a single partition, as was suggested by Partitionfinder 2.1.1 [22]. Phylogenetic analysis was performed using Bayesian inference (BI) and maximum likelihood (ML) analyses.

Bayesian inference was performed by running two parallel analyses in MrBayes 3.2.7a [23], with each run consisting of six Markov chains, and the chain temperature was set at 0.02 in all analyses. The convergences between runs (i.e., split deviation frequencies lower than 0.01) were reached after 0.5–1 million generations; therefore analyses were stopped after 5 million generations, and the sampling frequency was one tree per 1000 generations. Consensus trees were calculated after omitting the first 25% trees as burning. Maximum likelihood analyses were performed using RAxML 8.2.12 [24], and the robustness of the nodes was assessed using rapid bootstrapping with the majority-rule criterion automatic halt (autoMRE). All ML and BI analyses were performed on the Cipres Science Gateway [25]. All trees were rooted on *Catoscopium nigritum* (Hedw.) Brid. based on the tree topology, published by Cox et al. [26].

## 3. Results

### 3.1. Phylogenetic Results

The topologies of trees inferred from the single-gene datasets are congruent. In all three trees, accessions of target species were found to form a well-to-maximally supported clade within the highly supported but weakly resolved Aongstroemiaceae clade, but not close to any other lineage within it. Of note is that the morphology of the Portuguese specimens scarcely matches the morphology of any of the previously known members within the Aongstroemiaceae group. At the same time, the single original accession of *Dicranella humilis,* a species that resembles Portuguese plants morphologically, was found in the maximally supported grouping with *D. rufescens*, well outside the Aongstroemiaceae clade in all analyses. Of the three specimens of the enigmatic Portuguese plants involved in the molecular phylogenetic study (BF9, BF10, and BF11; see Appendix A), BF9 and BF11 have identical or nearly identical sequences of all three studied markers, while BF10 does not possess all molecular synapomorphies characteristic of the two other specimens.

**Figure 1:**
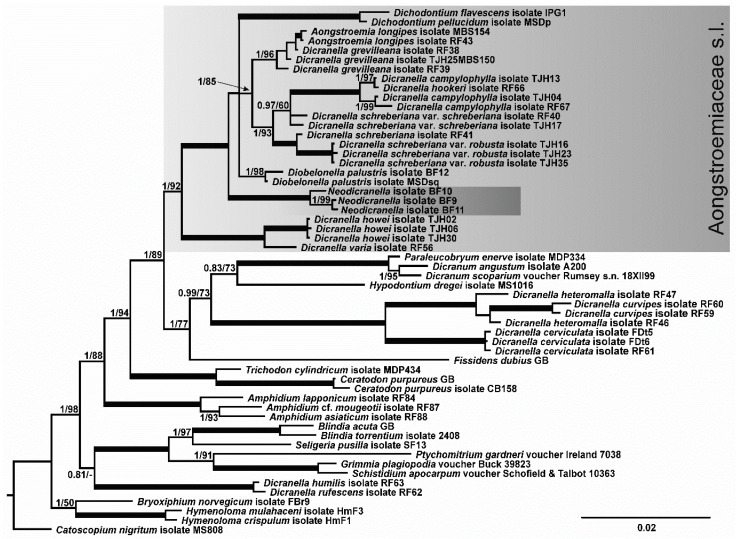
Bayesian tree of a selection of Dicranidae species, with particular focus on Aongstroemiaceae, inferred from combined sequences of cp *trnS-rps4* and *trnL-trnF* regions and the mt *nad5* intron. Posterior probabilities of ≥0.8 and bootstrap values inferred from ML of ≥50 are shown above branches; maximally supported nodes (PP = 1, BS = 100) are indicated by thick solid lines. The clade corresponding to Aongstroemiaceae s.l. cf. Bonfim-Santos et al., 2021 is highlighted by the gray box. For details, see Appendix A.

In the tree inferred from the combined *trnL-F*/*rps4*/*Nad5* dataset (Figure 1), the maximally supported Portuguese ‘*Dicranella* sp.’ clade occupies a sister position to the Aongstroemiaceae s.str. clade (sensu [4]), which is not supported, while the joint clade of ‘*Dicranella* sp.’ clade plus ‘Aongstroemiaceae s.str. clade’ has maximal support. Therefore, and somewhat unexpectedly, our phylogenetic reconstruction resolved the position of the Portuguese ‘*Dicranella* sp.’ outside the previously known groupings of *Dicranella*-like mosses. Moreover, although our analyses revealed familial assignment of the target species, they did not indicate any close relatives within Aongstroemiaceae.

### 3.2. Taxonomy

***Neodicranella*** R.D.Porley, Fedosov & Plášek, ***gen. nov.***(Aongstroemiaceae De Not.).

**Type:** *Neodicranella hamulosa* (see below).

**Diagnosis:** A combination of characters separates the new genus from other genera of the Dicranidae: small *Dicranella*-like plants; leaves lacking a sheathing base; costa excurrent in a subulate acumen with a dorsal and a ventral epidermis and a median stereid band; linear, smooth, thick-walled laminal cells; an inclined and short gibbous capsule; a Dicranoid peristome with small backward-pointing hooks on the distal segments; and rhizoidal tubers with slightly protuberant cells.

**Diagnosis:***Hoc compositum signorum novum genus ab alliis congeneribus Dicranidarum separat: plantae parvae Dicranellae similes; folia basi non vaginantia, e cellulis linearibus laevibusque parietibus crassis areolata; costa in subula evanida, in sectione transversali e tres stratis cellularum formata, ventrali dorsalique cellulis amplis epidermide, interno e cellulis parvis stereidarum; capsula inclinata brevisque gibbosa; peristomium dicranoideum, e dentibus bifidis, ramulis in parte distali hamulis retroflexis praeditis; tubera rhizoidalia e cellulis protuberantibus fabricata*.

**Etymology:** The name is derived from *Dicranella*, a group of small pioneer acrocarpous mosses in which the sole species of the new genus would be placed, had not the molecular phylogenetic study revealed its true affinity.

**Note:** The genus is introduced to accommodate a single species based on the result of the molecular phylogenetic study. For a description and differentiation, see the protologue of *N. hamulosa* below.

**Figure 2:**
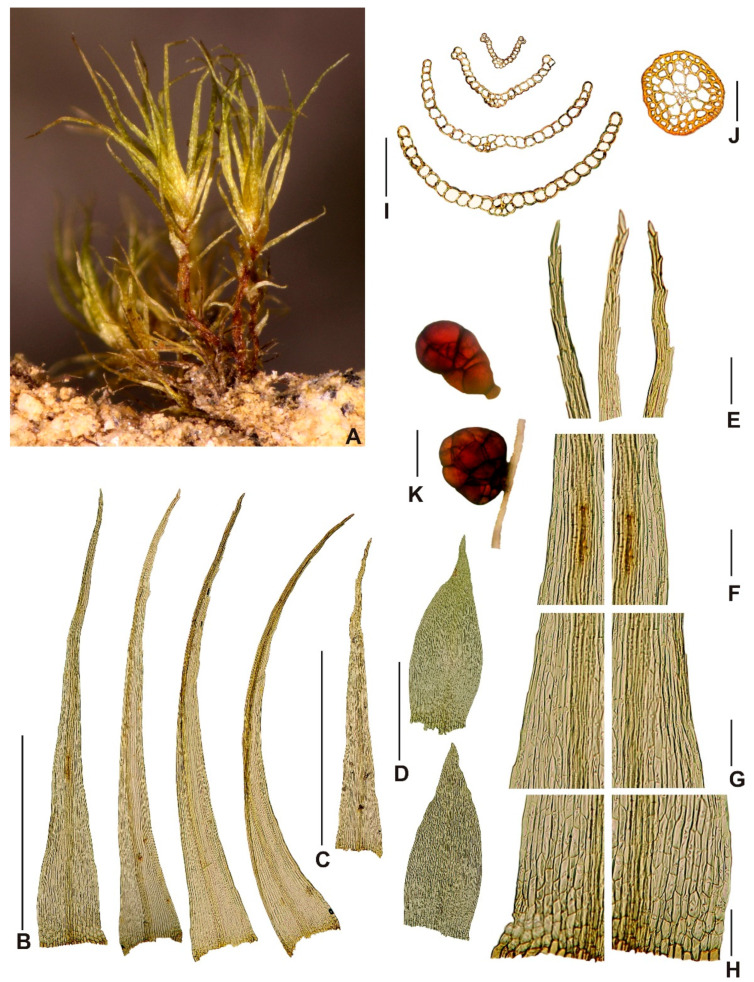
Macro and micro photographs of gametophyte of *Neodicranella hamulosa*: (**A**) view on sterile plants, (**B**) leaves from upper part of stem, (**C**) leaf of lower part of stem, (**D**) perichaetial leaves, (**E**) leaf apex, (**F**) upper-leaf cells, (**G**) middle-leaf cells, (**H**) basal cells, (**I**) leaf cross sections, (**J**) stem cross section, and (**K**) tubers. Scale bars: **B**,**C**—1 mm; **D**—0.5 mm; **E**–**H**—100 µm; **I**,**J**—50 µm; and **K**—100 µm. Photos **A**–**C** and **E**–**K** from holotype (KRAM B-260000) and **D** from paratype 1.

**Figure 3:**
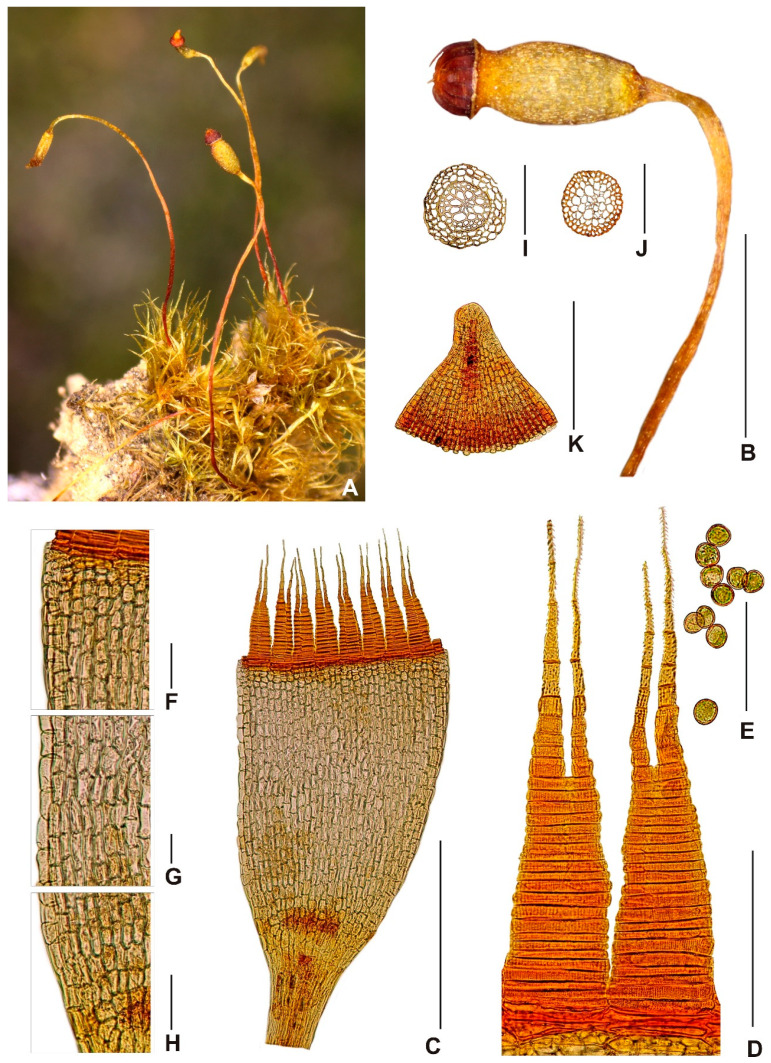
Macro and micro photographs of sporophyte of *Neodicranella hamulosa*: (**A**) view on fertile plants, (**B**) seta with capsule, (**C**) capsule with peristome, (**D**) detail view on peristome teeth, (**E**) spores, (**F**) upper-capsule cells, (**G**) middle-capsule cells, (**H**) basal-capsule cells, (**I**) cross section of basal part of seta with vaginula, (**J**) cross section of upper part of seta, and (**K**) operculum. Scale bars: **B**—1 mm; **C**—0.5 mm; **D**—100 µm; **E**,**H**—50 µm; **I**,**J**—250 µm; and **K**—0.5 mm. All photos from holotype (KRAM B-260000).

**Figure 4:**
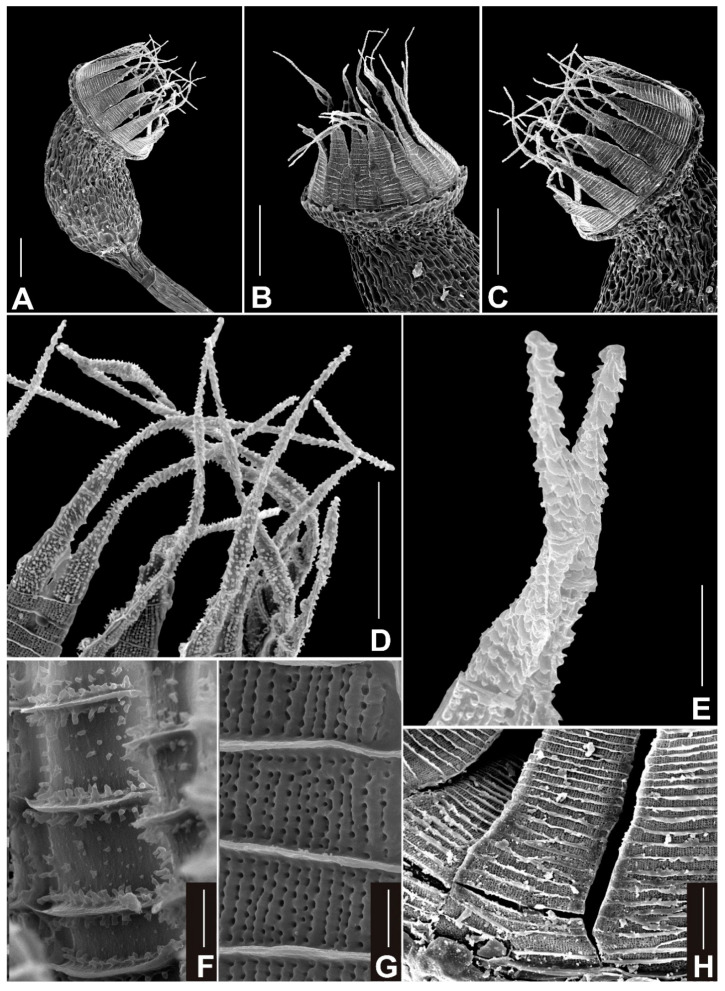
SEM photographs of capsule and peristome of *Neodicranella hamulosa*: (**A**) view of capsule, (**B**,**C**) views of peristome, (**D**,**E**) detailed view of upper part of outer peristome layer (OPL) densely ornamented with distinctive hamulose papillae, (**F**) detailed view of middle part of inner peristome teeth (IPL) showing conspicuous papillae on cross-trabeculae, (**G**) detailed view of middle part of outer peristome layer (OPL), and (**H**) basal part of outer peristome layer (OPL). Scale bars: **A**–**C**—100 µm; **D**—50 µm; **E**–**G**—10 µm; and **H**—50 µm. All photos from holotype (KRAM B-260000).

***Neodicranella hamulosa*** R.D.Porley, Fedosov & Plášek, ***sp. nov.*** (Figure 2, Figure 3 and Figure 4).

**Type: Portugal:** Faro District, Algarve, Vale das Amoreiras, near Aljezur, 37°20′12.49″ N 8°46′41.44″ W (WGS 84 UTM 29S 0,519,648 4132255), alt. 35 m, on the soil bank in N-facing *Quercus faginea* Lam. woodland, with *Cephaloziella divaricata* (Sm.) Schiffn. and *Pleuridium acuminatum* Lindb., 20 January 2019, *leg*. R.D.Porley (Holotype: KRAM B-260000; isotypes: MW [DNA Isolate BF10]), OSTR No. B3156, Hb. R.D.Porley.

Description: *Plants* small, yellowish green, when dry with slight luster, in turfs of intermingled stems, 3–5 mm tall. Stems c. 250 μm wide, occasionally branched below, in TS with a distinct central strand with 2–3 rows of enlarged, thin-walled medullary cells and a 1–2-layer cortex of smaller, thick-walled, yellowish cells; *rhizoids* scattered around the base of the stem, sometimes also above, brownish, fine rhizoids, straw colored, smooth, branched; *rhizoidal tubers* occasional, mostly translucent rufous brown, occasionally straw colored, sessile laterally or terminating rhizoids, spherical, pear shaped to ellipsoidal or irregular, multicellular with variably enlarged cells, slightly protuberant, variable in size, up to 320 μm long and 160 μm wide, but often much smaller; *leaves* erect spreading or subsecund when moist, flexuose when dry, upper leaves up to 2 mm long, narrowly triangular-lanceolate, 5–6 times as long as wide, widest at or near the base, tubulose when moist, gradually tapering to a narrow apex terminating in a single cell, subentire or bluntly serrulate subulate apex; *margins* plane throughout, in upper leaf bistratose in 1 row, occasionally 2 rows; *costa* faint, 25–38 μm at insertion, vanishing into longly acuminate subula, in TS weakly convex dorsally, with a median stereid band surrounded by larger epidermal cells; *laminal cells* rectangular to linear, prosenchymatous, non-porose, 9-10(-12) μm wide, (60-)80-110(-125) μm long, thick walled, 2.5 μm wide, yellowish, cells becoming narrower toward the apex, 5–7 μm wide, in TS laminal cells somewhat bulging, unistratose except at margins, basal angles with a few short rectangular-to-rounded cells with more pigmented walls, but scarcely forming a differentiated alar group; *dioicous*; *perigonia* terminal, gemmiform, perigonial leaves ovate, strongly concave, and abruptly subulate; *perichaetial leaves* differentiated from vegetative leaves, sheathing, 0.6–0.8 mm long, 0.28–0.32 mm wide, oblong, acute, costa ending below the apex, areolation similar to those in vegetative leaves, except cell walls thin, <2 μm wide; *setae* erect, reddish below grading to yellow above, 6–9 mm long, flexuose when dry, thin, ca. 120 μm diameter, sinistrose when dry, elongate cortical cells, 15–16 times as long as wide, vaginula brownish, bearing sparse hyaline hairs of 3–4 uniseriate cells, 75–95 μm long; *capsules* inclined, smooth, not sulcate when dry, pale green to straw colored, ovoid, gibbous, urceolate when dry and empty, up to 1 mm long, 0.6–0.7 mm wide, non-strumose; *operculum* reddish yellow, 0.4 mm, rostellate; *exothecial cells* oblong-rectangular (30-)35-50(-60) μm long, 15–25 μm wide, below the mouth 4–5 rows of quadrate to rounded cells on the lower side, longitudinal and transverse walls equally thickened; *stomata* few, at the base of the urn, bicellular; *annulus* weakly differentiated, not revoluble; *peristome teeth* 16, reddish orange below, yellow-orange distally, inserted just below the mouth, arising from a low basal membrane consisting of 3–5 rows of cells, extending ca. 22 μm above the mouth, teeth 55–57 μm wide at the base, 300–360 μm long, with a narrow irregular pellucid margin extending from the bifurcation to the base, divided to about halfway down into two segments of more or less equal length, filiform, ca. 160 μm long, tapering to a narrow subterete tip ca. 2.5 μm wide, the distal 5–7 segments densely ornamented with distinctive hamulose papillae, sharply backward-pointing hooks reminiscent of barbs, merging into stout conical papillae toward the base of the bifurcation, ultimate segment hyaline, more or less smooth, evanescent, the teeth below the bifurcation flat, somewhat uneven at margins, sharply trabeculate, with 18–24 trabeculae distinctly projected on the inner surface with conspicuous branched truncate or oblique papillae 2–2.5 μm high, dorsal plates between trabeculae with scattered conical papillae and vertically pitted-striate on both outer and inner surfaces, striae most distinct just above and below the bifurcation with fainter vertical striae on proximal plates; *spores* globose, (11-)12-16(-17) μm in diameter, greenish, minutely papillose; *calyptra* long cucullate, pellucid, straw colored ending in a dark mucro, smooth, fugacious.

Additional specimens examined (Paratypes): **(1) Portugal:** Faro District, Algarve, Vale das Amoreiras, near Aljezur, 37°20′11.70″ N 8°46′43.21″ W (UTM 29S 0,519,604 E 4,132,237 N), alt. 45 m, on open soil bank in N-facing *Quercus faginea* woodland, with *Cephaloziella divaricata*, 28 January 2021, *leg*. R.D.Porley (Hb. R.D.Porley); **(2)** *ibidem*, 37°20′11.24″ N 8°46′44.42″ W, (UTM 29S 0,519,577 4132227), alt. 42 m, on moderately steep ground in N-facing *Quercus faginea* woodland, with *Cephaloziella divaricata*, *C. turneri* (Hook.) Müll.Frib., *Ditrichum subulatum* Hampe, *Fossombronia angulosa* (Dicks.) Raddi, *Phymatoceros bulbicolosus* (Brot.) Stotler, W. T. Doyle & Crand.-Stotl. and *Sematophyllum substrumulosum* (Hampe.) E. Britton, 8 February 2021, leg. R.D.Porley (Hb. R.D.Porley); **(3)** Faro District, Algarve, Serra de Monchique, NE of Ginjeira, between Monchique and Picota, 37°18′38.86″ N 8°32′29.98″ W, (UTM 29S 0,540,615 4129447), alt. 523 m, on steep shaded bank of cutting in NE-facing *Castanea sativa* Mill. coppice in barranco, with *Epipterygium atlanticum* Hanusch, *Fissidens taxifolius* Hedw. and *Pogonatum aloides* (Hedw.) P. Beauv., 30 January 2017, *leg*. R.D.Porley (KRAM B-260001, Hb. R.D.Porley) [DNA isolate BF11]; **(4)** Faro District, Algarve, Serra de Monchique, N of Ginjeira, between Monchique and Picota, 37°18′43.06″ N 8°32′39.98″ W (UTM 29S 0,540,371 4129573), alt. 534 m, on humus of decomposing *Castanea* stool in NW facing *Castanea sativa* coppice, with *Fissidens viridulus* (Sw.) Wahlenb., 24 January 2017, *leg*. R.D.Porley (Hb. R.D.Porley); **(5)** Faro District, Algarve, Serra de Monchique, Ribeira de Seixe, near Barranco dos Pisões, 37°19′55.23″ N 8°34′05.41″ W, (UTM 29S 0,538,255 4131791), alt. 500 m, on moist rock by water in *Alnus glutinosa* (L.) Gaertn. riparium woodland, downstream of bridge, with *Epipterygium tozeri* (Grev.) Lindb., *Fissidens serrulatus* Brid. and *Lophocolea bidentata* (L.) Dumort., *Pogonatum aloides* and *Sematophyllum substrumulosum*, 22 February 2016, *leg*. R.D.Porley (Hb. R.D.Porley); **(6)** Beja District, Baixo Alentejo, Vale de Cova da Serra, near Relva Grande, 37°25′54.88″ N 8°37′15.54″ W, (UTM 29S 0,533,565 4142834), 215 m, on shaded streambank in *Rhododendron ponticum* subsp. *baeticum* (Boiss. & Reut.) Hand.-Mazz. ravine, with *Calypogeia fissa* (L.) Raddi, *Cephaloziella turneri*, *Ditrichum subulatum* and *Epipterygium atlanticum*, 17 May 2020, *leg*. R.D.Porley (KRAM B-260002, Hb. R.D.Porley) [DNA isolate BF9]. **(7)** *ibidem*, 37°25′54.54″ N 8°37′15.78″ W, (UTM 29S 0,533,521 4142846), 220 m, on bare ground with litter on N-facing barranco 30° slope, in *Quercus faginea* woodland, among a mixed mat of *Calypogeia fissa*, *Dicranella heteromalla* (Hedw.) Schimp., *Ditrichum subulatum*, *Entosthodon attenuatus* (Dicks.) Bryhn, *Fossombronia angulosa*, *Fissidens viridulus*, *Hypnum cupressiforme* Hedw. and *Kindbergia praelonga* (Hedw.) Ochyra, 12 April 2021, *leg*. R.D.Porley (Hb. R.D.Porley).

**Etymology:** The epithet of the new species alludes to the unique and conspicuous hamulose ornamentation of the bifid peristome teeth, from the Latin meaning *armed* or covered with small hooks resembling miniature barbs.

*Neodicranella hamulosa* in the Iberian Peninsula could be mistaken in the field for a number of other small dicranoid mosses, particularly in the absence of sporophytes, such as *Ditrichum subulatum* and *Pleuridium* sp. and other *Dicranella* s.l. species with non-sheathing leaves, including *D. howei* and *D. varia*. The immersed capsules of *Pleuridium* sp. immediately separate it, and a cross section of the leaves under the microscope reveals the broad nerve and partially bistratose lamina. The long subulate leaves of *Ditrichum subulatum* are more flexuose and wispy than those of *N. hamulosa*, and the capsule is straight with a short erect peristome, and under the microscope, the leaves in cross section show the wide nerve and bistratose lamina. The erect spreading to subsecund leaves of *Dicranella varia* also bears a resemblance to *N. hamulosa*, but the recurved margin of the former is characteristic, but this should be checked under the microscope. In addition, the wider nerve at the base (65–90 μm) and shorter basal cells (30–80 μm) separate *D. varia*. In addition, transverse sections of the costa *D. varia* reveal the presence of guide cells, and the capsules are markedly darker. The other *Dicranella* s.l. species that could be confused with *N. hamulosa*, *D. howei*, needs to be checked microscopically for a bistratose lamina, but in the field, it tends to have a darker-green color. *Dicranella heteromalla* is typically a larger plant, >1 cm, with falcate-secund leaves, shorter laminal cells (25–50 μm), wide nerve occupying c. 30% of leaf base, and capsules sulcate when dry, held on long yellowish setae.

**Figure 5:**
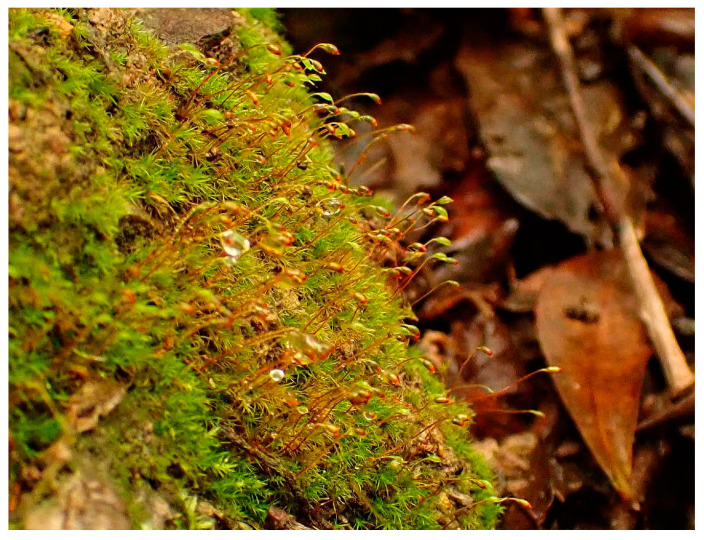
Habit of *Neodicranella hamulosa* in hydrated condition, on N-facing soil bank in *Quercus faginea* woodland, Vale das Amoreiras, Algarve, 28 January 2021 (paratype 1).

Rhizoidal tubers were found in all populations of *N. hamulosa*, although they can be sparse. They are too variable in morphology to be useful in identification, and are more or less similar to tubers in some former members of *Dicranella* s.l., but the somewhat protuberant cells distinguish them from others. Sporophytes appear to be frequent in *N. hamulosa*, observed between January and May, and enable certain identification. No other European *Dicranella* s.l. (or indeed, to the best of our knowledge, in the world) has the unique barbed appearance of the distal segments of the peristome teeth, as seen in *N. hamulosa* (see Figure 4). Although this is hardly discernible in the field, even with a x20 lens, the filiform tips of the distal peristome segments flex in a characteristic manner when dry, and with the delicately inclined gibbous capsules held on a thin seta < 1 cm long, red below merging to yellow above, together with yellowish erect leaves imparting a spikey appearance to the shoots, combine to suggest *N. hamulosa* may be close at hand (Figure 5).

## 4. Discussion

### 4.1. Taxonomical Notes

Based on the topology of phylogenetic reconstructions, we propose that the Portuguese plants essentially represent a new genus within the family Aongstroemiaceae, regardless of how broad or narrow the circumscription of this family is (i.e., with or without *D. varia* and *D. howei*). Although a comprehensive integrative taxonomic treatment of *Dicranella* is awaited, topologies of the obtained phylogenetic trees (in addition to trees presented by Bonfim-Santos et al. [4]) indicate there is no option of including the Portuguese plants in the genus *Dicranella*, since the type species, *D. heteromalla*, belongs to a different family. Furthermore, the Portuguese plants are also resolved outside of all known groupings of *Dicranella* species within Aongstroemiaceae, signifying a monotypic genus. Therefore, older generic names, which could be used for segregates of *Diranella* s.l., cannot be applied to our species.

Morphologically, both in gametophyte and sporophyte traits, the unknown *Dicranella*-like species from Portugal conform well to Aongstroemiaceae. In costal anatomy, the Portuguese plants resemble other *Dicranella* species found within Aongstroemiaceae s.l., such as *D. grevilleana* (Brid.) Schimp. and *D. schreberiana* (Hedw.) Hilf. ex H.A.Crum & L.E.Anderson, and also *Aongstroemia longipes* (Sommerf.) Bruch & Schimp. and *Diobelonella palustris*, while the costal anatomy is different in *Dichodontium pellucidum* (Hedw.) Schimp., *Dicranella varia*, and *D. howei*. Furthermore, in the absence of a sheathing leaf base, the Portuguese plants are clearly differentiated from *D. grevilleana*, *D. schreberiana*, and *Diobelonella palustris*. The rhizoidal gemmae (tubers) of the Portuguese plants show somewhat protuberant cells, differing from those of *D. grevilleana* and *D. schreberiana*, while morphologically similar tubers are reported in *Diobelonella palustris* [4]. Although the capsule morphology in the Portuguese plants is rather typical for Aongstroemiaceae, the backward-pointing papillae in the distal peristome segments, resembling spiked barbs (adj. hamulosus), represents a unique trait within the Aongstroemiaceae clade and possibly also within the Dicranidae.

**Figure 6:**
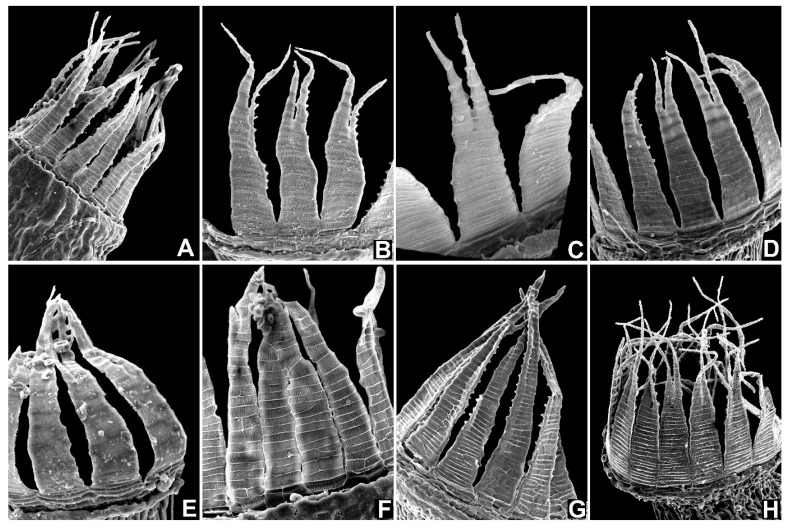
Comparison of peristomes in different species of *Dicranella* (**A**–**F**), *Diobelonella* (**G**), and *Neodicranella* (**H**) using SEM photographs: (**A**) *Dicranella grevilleana*, (**B**) *D. heteromalla*, (**C**) *D. howei*, (**D**) *D. schreberiana*, (**E**) *D. varia***,** (**F**) *D. humilis*, (**G**) *Diobelonella palustris*, and (**H**) *Neodicranella hamulosa*.

To investigate whether the hamulose distal peristome segment is indeed a unique trait, peristomes of a selection of other *Dicranella* species were examined by light microscopy and by SEM (Figure 6). Descriptions of *Dicranella* peristomes in standard floras are invariably vague, lacking in detail, often simply referring to 16 reddish teeth, divided to about halfway, papillose above and pitted striate below [5,6,27,28,29], yet our observations clearly showed that there is a considerable diversity of the peristome structure in the broadly conceived genus *Dicranella*. Even though this survey of the *Dicranella* peristome was limited, it covered all known European species, and if the peristome was not seen, the examination of the gametophyte or description of the species was sufficient for us to affirm that the Portuguese plants do not match any other known European *Dicranella*. Floras of other parts of the world, including those listed above and others [30,31,32,33] were also consulted, plus the revision of *Dicranella* in Brazil [8], but no species therein matches the present Portuguese species. In conclusion, based on the morphological evidence and on molecular evidence, *Neodicranella hamulosa* is a new hitherto undescribed species that represents an isolated phylogenetic lineage within the family Aongstroemiaceae, and we therefore established a new genus to accommodate it.

### 4.2. Habitat and Conservation

*Neodicranella hamulosa* was collected from forest biomes within the Mediterranean macrobioclimate region of southern Portugal, where warm wet winters and markedly dry summers are the norm. There is, however, a marked hyperoceanic influence attributable to the proximity of the North Atlantic Ocean, and advection fog can result in precipitation in the summer months. The Serra de Monchique is the most extreme south-west massif in continental Europe, and due to its approximately east–west orientation, the peaks and the surrounding foothills experience wide microclimatic variation with a sub-Mediterranean bioclimatic variant [16]. The maximum elevation, Fóia, reaches 902 m, and to the east is Picota, the second-highest peak at 774 m. Meteorological data are scarce, but the highest part of the massif receives over 1000 mm of annual rainfall, and the town of Monchique (395 m a.s.l.) an average of 1205 mm [34]. The area is geologically complex, with a wide variety of rocks of different ages, structures, and origins [35]. The central part of the massif, known as the Monchique Alkaline Complex, originated during the Upper Cretaceous, about 72 mya, by the intrusion of an igneous body upwelling through the older (Upper Carboniferous) sediments of the Brejeira Formation comprising schist and graywacke strata, which forms a rolling landscape typical of the surrounding foothills. The rocks of the central massif are mostly nepheline syenite [36], a silica under-saturated rock rich in alkalis and alumina and low in Mg and Fe, whereas the schist and graywackes are higher in quartz and lower in alumina and typically form shallow lithosoils.

**Figure 7:**
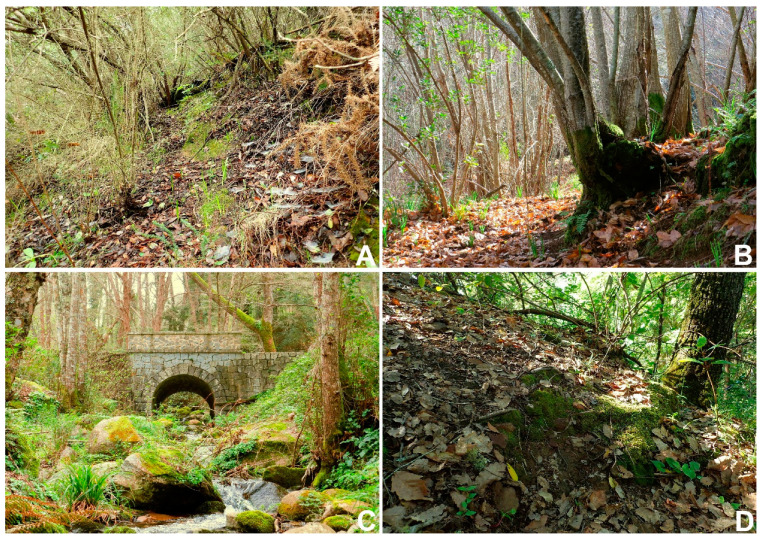
Habitat of *Neodicranella hamulosa* in (**A**) N-facing *Quercus faginea* woodland, Vale das Amoreiras, Algarve, 28 January 2021 (type locality); (**B**) in *Castanea sativa* coppiced woodland in NE-facing barranco, on soil bank (to right of picture), NE of Ginjeira, Serra de Monchique, Algarve, 30 January 2017 (paratype 3); (**C**) on boulder top in *Alnus glutinosa* riparian woodland, Ribeira de Seixe, Serra de Monchique, Algarve, 22 February 2016 (paratype 5); and (**D**) on soil bank in N-facing *Quercus faginea* woodland, Vale de Cova da Serra, Baixo Alentejo, 12 April 2021 (paratype 7).

At Vale das Amoreiras (Figure 7A) (type locality), *Neodicranella hamulosa* occurs on moderately steep N-facing ground over schist, mostly in partial gaps under a canopy of *Quercus faginea*, *Q. suber* L., *Erica arborea* L., and *Arbutus unedo* L. It occurs intimately mixed within a closed community of other bryophyte associates on soil, including *Cephaloziella divaricata*, *C. turneri*, *Ditrichum subulatum*, *Fissidens taxifolius*, *Fossombronia angulosa*, *Phymatoceros bulbicolosus*, and *Sematophyllum substrumulosum*, often with *Brachythecium rutabulum* (Hedw.) Schimp., *Kindbergia praelonga* (Hedw.) Ochyra, and nearby *Rhynchostegiella tubulosa* Hedenäs & J. Patiño. Other populations occur on patches of exposed soil with little more than *Cephaloziella divaricata*, *Pleuridium acuminatum*, and *Ditrichum subulatum* as companion species. The habitat and associated species are also quite similar at the Vale de Cova da Serra, where relict autochtonous *Quercus faginea* woodland survives on a north-facing slope (Figure 7D). Patches of sparsely vegetated ground are clearly a requirement for the maintenance of the *N. hamulosa* populations, and the omnipresence of wild boar (*Sus scrofa* L.) combined with thin soil over rock on slopes ensures the necessary disturbance is provided. Vale das Amoreiras is particularly notable for the abundance of *S. substrumulosum*; not only is it the dominant epiphyte, but it also occurs on rotten wood and cork and on soil and rock. *Sematophyllum substrumulosum* is a shade-tolerant thermophilic moss confined to regions in Europe with a strong Atlantic influence [37]. The occurrence of *S. substrumulosum* on all sites (except locality 4, a small relict less humid *Castanea* coppice) is notable, indicating that *N. hamulosa* may also be correlated with a warm humid bioclimate. *Castanea sativa* forests (Figure 7B) are also typically associated with areas that experience mild oceanic climates [38] and, since the 1820s, were planted over large areas of the Serra de Monchique [39]. However, disease, abandonment and fire have reduced their cover to a few relict fragments mostly on the north slopes of Fóia and on Picota. *Alnus glutinosa* riparian woodland is widespread in barrancos on the Serra de Monchique, and along the Ribeira de Seixe, *N. hamulosa* was growing on rock (Figure 7C) with *Fissidens serrulatus*, a sciophyte with a strong Mediterranean–Atlantic element. In addition, on rock at this locality is the red-listed *Campylostelium strictum* Solms, a plant also indicative of humid niches with a center of distribution on Macaronesia and west Mediterranean. It is notable, therefore, that a shared and consistent feature of *N. hamulosa* sites is relatively high humidity maintained throughout the year, either on predominately N-facing wooded slopes or by streams in sheltered barrancos.

**Figure 8:**
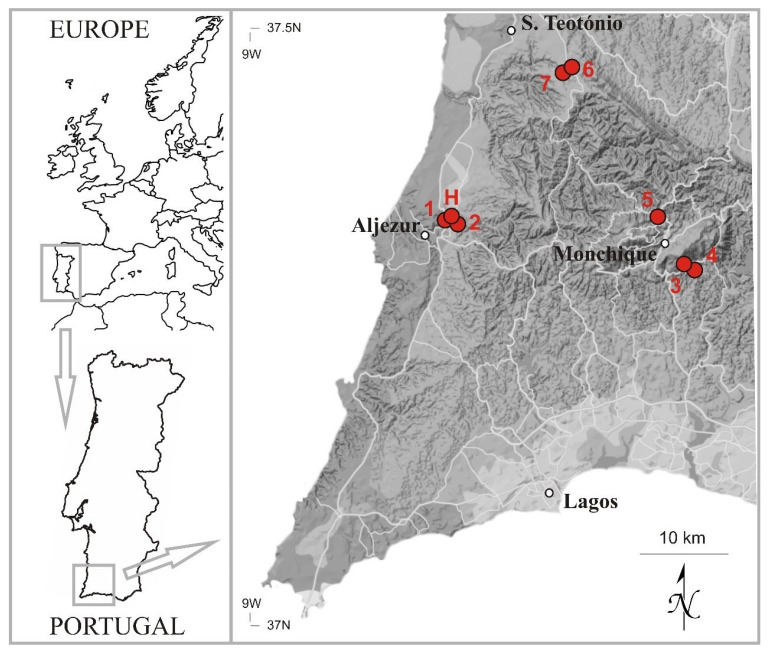
Distribution of *Neodicranella hamulosa* in south-west Portugal: (**H**) holotype and **(1–7)** paratypes (for details, see Section 3.2).

The currently known localities for *Neodicranella hamulosa* are widely scattered, albeit within a relatively small area of south-west Portugal (Figure 8), in the Monchiquense district [16]. Three of the localities are on the central massif of the Serra de Monchique, Algarve: two in the saddle between the towns of Monchique and Picota (523 m and 534 m a.s.l.) and one locality on Ribeira de Seixe (500 m a.s.l.). The other two localities are situated on the shales, one further west near Aljezur, Algarve (35–45 m a.s.l.), and the other just beyond the Algarve border to the north, in Baixo Alentejo (215–220 m a.s.l.). All localities are within the 76,000 ha Monchique Natura 2000 Special Area of Conservation (SAC), except for Vale das Amoreiras, which lies about 1.5 km outside the NW boundary of the SAC. It is nevertheless a proposed Área Protegida Privada and is currently under favorable management. However, despite the protection conferred by the SAC designation, commercial eucalyptus plantations, mostly of *Eucalyptus globulus* Labill., a non-native tree that is highly invasive and can seed into even the deepest barrancos, are extensive and planting continues apace on the Serra de Monchique, posing a serious threat to the remaining semi-natural habitats where *N. hamulosa* exists.

The impact of climate change on *N. hamulosa* cannot be anticipated with any certainty, although it is the implication for the habitat that may be the critical factor. It is predicted that *Quercus faginea* forests and the tertiary relic *Q. canariensis* would be negatively impacted if there is a shift to drier conditions [40], a forest type that has already seen a dramatic reduction in extent in Portugal since early times. Catastrophic wildfires are also an enormous issue on the Serra de Monchique and in the Mediterranean in general and are predicted to increase in severity and frequency [41]. The impact of fire is compounded by *Eucalyptus* monocultures and poor forestry management. There is virtually no part of the Serra de Monchique and the surrounding area that at some time has not been burnt, often multiple times. All localities where *N. hamulosa* was found show fire damage, evidenced by scorched trees and shrubs. At the Vale de Cova da Serra, *N. hamulosa* occurs in a humid *Rhododendron* ravine with *Epipterygium atlanticum*, at the bottom of a barranco (valley) with *Quercus faginea* woodland on the north-facing slope. *Rhododendron ponticum* subsp. *baeticum* is a tertiary relic from a period when a subtropical climate was predominant in the Iberian Peninsula, but as the climate gradually changed to a Mediterranean one, *R. ponticum* subsp. *baeticum* became confined to a few isolated refugia, including the Serra de Monchique. The vulnerability of Mediterranean deciduous forests is recognized by inclusion on Annex I of the EU 1992 Habitats Directive requiring SAC designation, including *Quercus faginea* and *Quercus canariensis* Iberian woods (Natura 2000 code 9240), *Castanea sativa* woods (9260), and *Rhododendron* ravines are included in Riparian formations on intermittent Mediterranean courses (92B0).

The Iberian Peninsula was one of the most important Pleistocene glacial refuges in the European subcontinent [42]. Médail and Diadema [43] analyzed the phylogeographical patterns of vascular plants, and the Algarve was identified as one of the 52 putative glacial refugia in the Mediterranean basin, although the Serra de Monchique was not highlighted as one of the 10 regional hotspots of plant biodiversity. Gómez and Lunt [44] reviewed the phylogeographic and biogeographic evidence of multiple and isolated glacial refugia and refuted the idea of the entire peninsula as a continuous Pleistocene refuge. The wide range of climate types and the occurrence of mountain ranges with predominantly east–west orientation provided opportunities for a range of species to survive adverse climate periods and undergo genetic differentiation. A refugium for white oaks, including *Quercus faginea* and *Q. canariensis*, based on the analysis of DNA haplotypes has been identified in the southwest peninsula [45], an area that encompasses the Serra de Monchique.

The level of endemism in bryophytes is typically low compared to vascular plants [46], though the list of endemics for any particular area is fluid as field exploration and taxonomic studies progress. In the Iberian Peninsula, there are currently 15 Iberian endemic bryophytes, including *Neodicranella hamulosa*. In mainland Spain, there are five endemics (updated and adapted from Albertos et al. [47] and Hodgetts and Lockhart [13]), and in continental Portugal, there are currently three known endemics, *Racomitrium lusitanicum* Ochyra & Sérgio; *Coscinodon monchiquensis* R.D.Porley, Ochyra & Ignatova (Serra de Monchique [48]); and *N. hamulosa* (this paper), representing about 0.4% of the bryophyte flora, a level more or less comparable to Spain [49]. Moreover, *N. hamulosa* would appear to be the first report of an endemic bryophyte genus to the Iberian Peninsula.

At the Vale das Amoreiras and the Vale de Cova da Serra, *Quercus canariensis* and *Q. marianica* are companion species in the *Q. faginea* forest; such thermophilous marcescent formations are characteristic of the ecotone between temperate areas with cold winters and mild rainy summers and Mediterranean areas with dry and hot summers. These transitional areas are vital refuges for endemics and species from contrasting zones when environmental changes cause range shifts [50]. In the past, even prior to the Late Glacial Maximum (ca. 22Ky), the entire Monchiquense district supported forest characterized by oaks from sect. *Quercus*, including *Q. canariensis* and *Q. faginea* (and their hybrid *Q. marianica)*. *Quercus canariensis* requires higher precipitation (>800 mm/year) than *Q. faginea* and today is confined to deep barrancos and other situations receiving advection fog [50,51]. *Quercus suber* (cork oak) is also a member of these forests, but it is more characteristic of drier sites on the higher rocky slopes on shallow lithosols. It is conceivable that *N. hamulosa* was more widespread in the past and that its destiny may be closely linked to the fate of the sub-Mediterranean marcescent forest, which, to quote De Rios et al. [40], is gloomy.

## Data Availability

All authors agree with MDPI Research Data Policies.

## Appendix A

Specimen voucher information and *nad5*, *trnS-rps4 spacer/rps4 gene*, *trnL gene/trnL-trnF* spacer GenBank accession numbers. Newly obtained sequences supplied with voucher information are provided in bold. The source GenBank is indicated for sequences downloaded from Genbank for which the geographic origin, collector, and collector number and herbarium were not indicated. Sequences missing from the dataset are marked with “–”.

*Amphidium asiaticum* Sim-Sim, Afonina & M. Stech **RF88** MN092574, MN092440, MN092377;

*Amphidium lapponicum* (Hedw.) Schimp. **RF84** MN092571, MN092437, MN092374;

*Amphidium mougeotii* (Bruch & Schimp.) Schimp. **RF87** MN092573, MN092439, MN092376;

*Aongstroemia longipes* (Sommerf.) Bruch & Schimp. **MBS154** MN177982, MN187470, MN178048; **RF43** MN177984, MN187471, MN178050;

*Blindia acuta* (Hedw.) Bruch & Schimp. Genbank AY908928, JQ890483, KX387230;

*Blindia torrentium* Cardot & Broth. **2408** –, MN718533, MN718472;

*Bryoxiphium norvegicum* (Brid.) Mitt. **FBr9** MN092589, MN092455, MN092386;

*Catoscopium nigritum* (Hedw.) Brid. Genbank AY908927, AF491051, AF497128;

*Ceratodon purpureus* (Hedw.) Brid. Genbank AY908862, AB848717, AB848718;

*Ceratodon purpureus* (Hedw.) Brid. **MSCp** KX580395, KX580500, AF135096;

*Dichodontium flavescens* (Dicks. ex With.) Lindb. **IPG1** –, MN187479, MN178059;

*Dichodontium pellucidum* (Hedw.) Schimp. **MSDp** MN177991, MN187480, MN178060;

*Dicranella campylophylla* (Taylor) A.Jaeger **TJH04** MN177992, MN187481, MN178061; **TJH13** MN177993, MN187482, MN178062; **RF67 R. Smith 2763 (LE, as *D. cardotii*)** –, MW881244, MW881241;

*Dicranella cerviculata* (Hedw.) Schimp. **FDt5** MN177995, MN187483, MN178063; **FDt6** MN177996, MN187484, MN178064; **RF61** MN177997, MN187485, MN178065;

*Dicranella curvipes* (Lindb.) Ignatov **RF59** MN178002, MN187491, MN178072; **RF60** –, MN187492, MN178073;

*Dicranella grevilleana* (Brid.) Schimp. **RF38** MN178003, MN187493, MN178074; **RF39** MN178004, MN187494, MN178075; **TJH25/MBS150**, MN178006, MN187496, MN178077;

*Dicranella heteromalla* (Hedw.) Schimp. **RF46** –, MN187497, MN178078; **RF47** MN178007, MN187498, MN178079;

*Dicranella hookerii* (Müll. Hal.) Cardot **RF66 Greene 2988 (LE)** –, MW881243, MW881240;

*Dicranella howei* Renauld & Cardot **TJH02** MN178010, MN187501, MN178082; **TJH06** –, MN187502, MN178083; **TJH30** MN178011, MN187503, MN178084;

*Dicranella rufescens* (With.) Schimp. **RF62** MN178013, MN187506, MN178087;

*Dicranella humilis* Ruthe **RF63** MN178014, MN187507, MN178088 (as *D. rufescens*);

*Dicranella schreberiana* (Hedw.) Hilf. ex H.A.Crum & L.E.Anderson var. schreberiana **TJH17** MN178015, MN187508, MN178089; **RF40** –, MW881242, MW881239;

*Dicranella schreberiana* var. *robusta* (Schimp. ex Braithw.) H.A.Crum & L.E. Anderson **RF41** MN178016, MN187509;

*Dicranum angustum* Lindb. **A200** MN092630, MN092497, KT580676;

*Dicranum scoparium* Hedw. **Rumsey s.n. 18XII99** AY908884, AF234158, KF424001;

*Diobelonella palustris* (Dicks.) Ochyra MSDsq KX580424, KX580510, AF135090; BF12 Shikotan Island, voucher Bakalin VGBI99275, dupla MW MW798728, MW798724, MW798731;

*Fissidens dubius* P. Beauv. Genbank JX241619, AF231281, FJ572491;

*Grimmia plagiopodia* Hedw. Genbank AY908919, AY908144, AJ879761;

*Hymenoloma crispulum* (Hedw.) Ochyra **HmF1** KX369287, KX369279, MN092411;

*Hymenoloma mulahaceni* (Höhn.) Ochyra **HmF3** MN092412, KX369280, KX369289;

*Hypodontium dregei* (Hornsch.) Müll.Hal. **MS1016** KX580414, KX580518, JQ690733;

*Neodicranella hamulosa* R.D.Porley, Fedosov & Plášek BF9, Portugal, voucher Porley 17.V.2020 MW798725, MW798721, MW798729; BF10, Portugal, voucher Porley 20.I.2019 MW798726, MW798722, –, BF11, Portugal, voucher Porley 30.I.2017 MW798727, MW798723, MW798730;

*Paraleucobryum enerve* (Thed.) Loeske **MSPe** MN178042, MN187536, AF135075/AF136083;

*Schistidium apocarpum* (Hedw.) Bruch & Schimp. Genbank AY908920, JQ040708, GQ428079;

*Seligeria pusilla* (Hedw.) Bruch & Schimp. **SF13** KR026971, KR026960, KX387262;

*Trichodon cylindricus* (Hedw.) Schimp. **MDP434** AY908863, AY908125, KX446935.
